# The Chronic Kidney Disease-Epidemiology Collaboration (CKD-EPI) equation does not improve the underestimation of Glomerular Filtration Rate (GFR) in people with diabetes and preserved renal function

**DOI:** 10.1186/s12882-015-0196-0

**Published:** 2015-12-03

**Authors:** Richard J. MacIsaac, Elif I. Ekinci, Erosha Premaratne, Zhong X. Lu, Jas-mine Seah, Yue Li, Ray Boston, Glenn M. Ward, George Jerums

**Affiliations:** Department of Endocrinology and Diabetes, St Vincent’s Hospital Melbourne, 4th Floor, Daly Wing, 35 Victoria Parade, PO Box 2900, Fitzroy, VIC 3065 Australia; Endocrine Centre, Austin Health, West Heidelberg, 3081 Victoria Australia; Menzies School of Health Research, Casuarina, 0811 Northern Territory Australia; Melbourne Pathology, Collingwood, 3066 Victoria Australia; Clinical Chemistry, St Vincent’s Hospital Melbourne, Fitzroy, 3065 Victoria Australia; Department of Medicine, St Vincent’s Hospital, University of Melbourne, Fitzroy, 3065 Victoria Australia; Department of Medicine, Austin Health, University of Melbourne, Heidelberg, 3084 Victoria Australia

**Keywords:** Diabetes, Chronic, Kidney Disease, Nephropathy, CKD-EPI equation, Glomerular filtration rate

## Abstract

**Background:**

Our hypothesis was that both the Chronic Kidney Disease-Epidemiology Collaboration (CKD-EPI) and Modification of Diet in Renal Disease (MDRD) equations would underestimate directly measured GFR (mGFR) to a similar extent in people with diabetes and preserved renal function.

**Methods:**

In a cross-sectional study, bias (eGFR – mGFR) was compared for the CKD-EPI and MDRD equations, after stratification for mGFR levels. We also examined the ability of the CKD-EPI compared with the MDRD equation to correctly classify subjects to various CKD stages. In a longitudinal study of subjects with an early decline in GFR i.e., initial mGFR >60 ml/min/1.73 m^2^ and rate of decline in GFR (ΔmGFR) > 3.3 ml/min/1.73 m^2^ per year, ΔmGFR (based on initial and final values) was compared with ΔeGFR by the CKD-EPI and MDRD equations over a mean of 9 years.

**Results:**

In the cross-sectional study, mGFR for the whole group was 80 ± 2.2 ml/min/1.73 m^2^ (*n* = 199, 75 % type 2 diabetes). For subjects with mGFR >90 ml/min/1.73 m^2^ (mGFR: 112 ± 2.0, *n* = 76), both equations significantly underestimated mGFR to a similar extent: bias for CKD-EPI: -12 ± 1.4 ml/min/1.73 m^2^ (*p* < 0.001) and for MDRD: -11 ± 2.1 ml/min/1.73 m^2^ (*p* < 0.001). Using the CKD-EPI compared with the MDRD equation did not improve the number of subjects that were correctly classified to a CKD-stage. No biochemical or clinical patient characteristics were identified to account for the under estimation of mGFR values in the normal to high range by the CKD-EPI equation. In the longitudinal study (*n* = 30, 66 % type 1 diabetes), initial and final mGFR values were 102.8 ± 6 and 54.6 ± 6.0 ml/min/1.73 m^2^, respectively. Mean ΔGFR (ml/min/1.73 m^2^ per year) was 6.0 by mGFR compared with only 3.0 by MDRD and 3.2 by CKD-EPI (both *p* < 0.05 vs mGFR)

**Conclusions:**

Both the CKD-EPI and MDRD equations underestimate reference GFR values >90 ml/min/1.73 m^2^ as well as an early decline in GFR to a similar extent in people with diabetes. There is scope to improve methods for estimating an early decline in GFR.

## Background

It is well recognised that estimating GFR using the Modification of Diet in Renal Disease (MDRD) equation significantly underestimates reference GFR values in the normal-high range in people with and without diabetes [1]. In an attempt to overcome some of the limitations of the MDRD equation, the Chronic Kidney Disease-Epidemiology Collaboration (CKD-EPI) equation has been developed, using the same variables as the MDRD equation. In particular, it has been reported to reduce bias compared to the MDRD equation for GFR > 60 ml/min/1.73 m^2^ in various study populations [2, 3]. This reduction in bias has been attributed to the characteristics of the populations from which the CKD-EPI and the MDRD equations were derived, with the mean measured GFR levels being 68 and 40 ml/min/1.73 m^2^ in these respective populations [1, 4]. Unlike the MDRD equation, the CKD-EPI equation also contains a spline term for serum creatinine (at 62 μmol/L for females and 80 μmol/L for males) to account for the weaker relationship between serum creatinine and GFR at lower compared with higher creatinine levels [4, 5].

Despite the above, the CKD-EPI equation appears to still significantly underestimate normal to high GFR levels to a similar extent as the MDRD equation in people with diabetes [6–9]. There is also evidence to suggest that the CKD-EPI equation underperforms in people with diabetes compared to those without diabetes [10]. One of potential limitations of many of the above studies that have assessed the performance of the CKD-EPI equation in diabetes is that they have not included a direct measurement of serum creatinine by an enzymatic method, the accepted ideal method for measuring serum creatinine. Furthermore, few previous studies have included a longitudinal assessment of the bias observed with estimating GFR by the CKD-EPI equation and hence the ability of this method to accurately assess an early decline in GFR. In people with diabetes, the reasons for the apparent lack of improvement in the underestimation of reference GFR levels when an eGFR is derived from the CKD-EPI compared with the MDRD equation also remain unclear.

Given the above, our hypothesis was that the CKD-EPI and MDRD equations would underestimate directly measured GFR to a similar extent in people with diabetes who have GFR values in the normal to high range. Therefore, the aim of this study was to compare the performance of the CKD-EPI and MDRD equations in people with diabetes for estimating directly measured GFR (mGFR) values, when measurements of serum creatinine are based on an enzymatic assay. Furthermore, we examined the ability of the CKD-EPI compared with the MDRD equation to correctly classify subjects to various CKD stages. We also explored whether certain clinical or biochemical characteristics of subgroups of patients with diabetes influenced the performance of the CKD-EPI equation. Finally, we assessed the ability of the CKD-EPI equation to accurately monitor early longitudinal changes in GFR.

## Methods

### Study population and design

Subjects involved in this study attended the diabetes clinics at Austin Health, University of Melbourne affiliated tertiary referral centre. Initially, a cross-sectional study of 199 consecutive patients who had an isotopic GFR (mGFR) measurement was performed followed by a separate longitudinal study of 30 patients with diabetes that were identified as having an early decline in GFR (rate of mGFR decline > 3.3 ml/min/1.73 m^2^ per year) and who were followed for nine years [11, 12]. As previously described, this approach allowed us to follow a group of patients whose renal function declined from a normal-high GFR to subnormal mGFR (17). The rate of decline in mGFR was based on the difference between the first and last mGFR over the nine year interval.

Subjects with known non-diabetic renal disease were excluded. A fasting blood sample was collected on the morning of the mGFR estimation for the measurement of glucose, electrolytes, glycated haemoglobin (HbA1c) and lipid levels. The following clinical information was recorded for all patients: age, gender, type and duration of diabetes, height, weight, body mass index (BMI), albumin excretion rate (AER; average result of at least two 24-h urine collections), blood pressure and smoking history, history of retinopathy and history of any macrovascular diseases classified as coronary artery disease, stroke or peripheral vascular disease. The type of diabetes was classified according to World Health Organization criteria. The research reported in the manuscript was carried out according to the declaration of Helsinki (2000) of the World Medical Association, and was approved by the Human Research Ethics Committee of Austin Health. All participants provided written consent.

### Laboratory methods

The reference mGFR measurement was obtained using a plasma decay technique for samples collected at 120, 165 and 210 min following a single bolus injection of ^99m^Tc-diethylene-triamine-pentaacetic acid (DTPA). The Brochner–Mortensen correction was then applied to the decay results obtained by this single bolus injection disappearance method [13, 14]. Plasma glucose, electrolytes, lipids, HbA1c levels and urinary albumin were measured as described previously [15].

Creatinine in the cross-sectional analysis was measured with the Roche enzymatic assay which is traceable to the Isotope Dilution Mass Spectrometry (IDMS) method as described previously. The longitudinal study was based on an analysis of serum creatinine levels that were recorded in a clinic database where creatinine levels had been measured by the modified Jaffe reaction on a Hitachi 911 automatic analyser (Roche Diagnostics, Mannheim, Germany). The intra-assay coefficient of variation (CV) was 2.3 % at a creatinine concentration of 67.1 mol/L for this assay. There was an excellent linear correlation between all 199 sample assayed by the enzymatic method for the cross sectional analysis and the above Jaffe method (y = 0.953x + 0.767, r^2^ = 0.965) across a wide range (25 to 250 mmol/L) of creatinine values. However, creatinine values assayed by the enzymatic method were slightly lower compared to the Jaffe method. The creatinine values used for the longitudinal analysis were therefore directly calibrated with the enzymatic method based on the ratio of creatinine values measured by both the Jaffe and enzymatic methods. In the 199 serum samples from the cross-sectional analysis we derived a recalibrated enzymatic creatinine value that = 0.961 × Jaffe measured creatinine.

Estimations of GFR based on serum creatinine were derived from the “175” MDRD equation and the CKD-EPI equation as outlined below-

The adjusted “175” MDRD-4 variable equation (for creatinine measurements traceable to the IDMS method ) i.e., eGFR = 175 × [(S_cr_ × 0.0113)^-1.154^] × (age)^-0.203^ × (0.742 if female) × (1.212 if African-American).

The CKD-EPI equation i.e., eGFR = 141 × min(Scr × 0.0113/k, 1)^α^ × max(Scr × 0.0113/k, 1)^-1.209^ × 0.993^Age^ × 1.018 [if female] × 1.159 [if black], where Scr is serum creatinine, k is 0.7 for females and 0.9 for males, ^α^ is -0.329 for females and -0.411 for males, min indicates the minimum of Scr/k or 1, and max indicates the maximum of Scr/k or 1.

### Statistical analysis

The agreement between mGFR and eGFR (derived from either the MDRD or CKD-EI equations) was estimated using the Concordance Correlation Coefficient (rho_c) which measures both precision and accuracy to determine how far the difference between the observed data deviate from the line of perfect concordance [16]. When describing the strength of agreement, according to the commonly adopted classification, a rho_c <0 = poor agreement, 0-0.2 = slight, 0.21-0.4 = fair, 0.41-0.6 = moderate, 0.61-0.8 = substantial and 0.81-1 = almost perfect [17]. The Bland-Altman plot method was also used to test the agreement between mGFR and an eGFR derived from the MDRD or the CKD-EPI equations for the entire study population in the cross-sectional analysis [18]. The performance of creatinine based eGFR equations was also compared by assessment of bias (eGFR - mGFR), so that bias is a negative value when eGFR underestimates mGFR, accuracy (the proportion of results falling within 15 % (P15) or 30 % (P30) of mGFR values) and precision, expressed as standard deviation (SD) of the bias [19]. In addition, the proportion of patients classified into the correct CKD stage according to mGFR measurement by each prediction equation (MDRD or CKD-EPI) was also determined. The proportion correctly classified was calculated for the whole study population as well as for each CKD stage separately (stage 1: ≥ 90, stage 2: 60-89 and < stage 3: < 60 ml/min/1.73 m^2^). Results are expressed as mean ± SD and statistical analysis of continuous variables was performed using ANOVA with the Tukey test for multiple comparisons or by unpaired t-tests where appropriate. Categorical variables were compared using Chi-χ^2^ analysis. Data confirmation for normality prior to statistical tests and in conjunction with confidence intervals was performed using the Shapiro Wilks test (Stata 13.1). A P-value of 0.05 was used in conjunction with all statistical tests to distinguish significant outcomes or differences.

## Results

### Cross-sectional analysis

The characteristics of the study population used for the cross sectional study (*n* = 199, 75 % type 2 diabetes), and divided into strata of mGFR > 90 (*n* = 76), 60-90 (*n* = 63) and < 60 (*n* = 60) ml/min/1.73 m^2^ are shown in Table 1. As expected, patients with the highest mGFR levels were younger and more likely to have type 1 diabetes, shorter duration of diabetes, a lower AER, higher rate of normoalbuminuria, lower systolic blood pressure and lower rate of macrovascular complications than those with lower mGFR levels. HbA1c and fasting blood glucose levels were higher in patients with higher mGFR levels. There were no differences in the rates of use of renin-angiotensin inhibiting agents or fenofibrate, medications known to alter creatinine or eGFR levels, in patients with various mGFR levels.

**Table 1:** Patient characteristics for the cross-sectional analysis (*n* = 199). Values shown as mean ± SD or median (interquartile range) and p values obtained from comparison between the 3 mGFR groups, calculated from a one-way ANOVA or χ^2^ test

Parameter	All (n=199)	mGFR <60 (n=60)	mGFR 60-90 (n=63)	mGFR >90 (n=76)	*p* value
Age (Yr)	62.8 ± 12.7	70.9 ± 10.8	65.4 ± 9.2	54.3 ± 11.3	<0.001
Gender (% males)	67	65	65	70	0.79
Type diabetes (% T1/T2)	25/75	15/85	21/79	37/63	0.009
Duration of diabetes (Years)	18.0 ± 11.4	20.9 ± 12.0	19.1 ± 12.7	14.8 ± 8.9	0.007
Height (cm)	168.6 ± 9.3	167.5 ± 9.2	167.5 ± 9.6	170.5 ± 8.9	0.12
Weight (kg)	88.8 ± 21.1	85.8 ± 19.6	89.3 ± 24.5	90.7 ± 19.3	0.41
BMI (kg/m^2^)	31.1 ± 7.0	30.2 ± 6.0	31.8 ± 8.5	31.1 ± 6.1	0.46
mGFR (ml/min/1.73m^2^)	80.0 ± 30.9	44.2 ± 10.8	75.5 ± 9.8	111.8 ± 17.0	<0.001
MDRD (ml/min/1.73m^2^)	78.4 ± 28.2	48.2 ± 13.3	79.5 ± 17.5	100.8 ± 22.5	<0.001
CKD-EPI (ml/min/1.73m^2^)	78.4 ± 24.9	49.5 ± 14.2	80.7 ± 15.2	99.4 ± 13.1	<0.001
AER (μg/min)	10.4 (4.3, 26.3)	20.7 (7.6, 71.3)	5.8 (3.5, 19.1)	8.6 (4.3, 20.7)	0.47
AER (% normo/micro/macro)	69.2/24.7/6.1	54.2/32.2/13.6	69.8/23.8/6.4	80.3/19.7/0	0.004
HbA_1C_ (%)	7.60 ± 1.1	7.35 ± 1.0	7.42 ± 0.085	7.94 ± 1.3	0.002
HbA_1C_ (mmol/mole)	60	57	58	63	0.002
Systolic blood pressure (mmHg)	131.9 ± 14.6	135.2 ± 17.4	132.7 ± 15.2	128.7 ± 10.8	0.03
Diastolic blood pressure (mmHg)	71.4 ± 10.1	67.9 ± 9.6	72.2 ± 10.9	73.5 ± 9.2	0.005
Any blood pressure therapy (%)	81	88	82	76	0.21
ACE inhibitor therapy (%)	47	52	42	46	0.65
Lipid lowering therapy (%)	83	88	89	76	0.08
Fenofibrate therapy (%)	8	11	8	5	0.55
Retinopathy ()	53	61	51	48	0.34
IHD (%)	31	39	38	19	0.02
CVD (%)	5	5	7	3	0.56
PVD (%)	13	25	12	4	0.002
History of macrovascular disease (%)	40	49	48	23	0.002

*BMI* Body Mass Index, *mGFR* measured Glomerular Filtration Rate, *MDRD* Modification of Diet in Renal Disease, *CKD-EPI* Chronic Kidney Disease-Epidemiology Collaboration, *AER* Albumin Excretion Rate, *Normo* Normoalbuminuria, *Micro* Microalbuminuria, *Macro* Macroalbuminuria, *HbA1c* Glycated Haemoglobin, *ACE* Angiotensin Converting Enzyme, *IHD* Ischaemic Heart Disease, *CVD* Cerebrovascular Disease, *PVD* Peripheral Vascular Disease

**Fig. 1:**
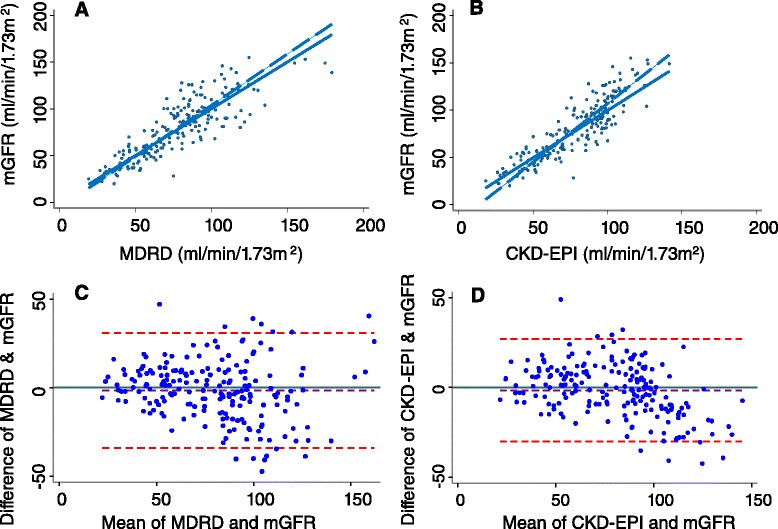
Concordance between eGFR derived from the MDRD (Panel **a**) or CKD-EPI (Panel **b**) equations with mGFR in the entire study population (*n* = 199). The reduced major axis (the actually agreement between eGFR and mGFR) is shown as the broken line and the line of perfect concordance, i.e., if there was perfect agreement between mGFR and eGFR is shown by the solid line. Bland-Altman plots summarising the agreement of an eGFR derived from the MDRD (Panel **c**) or CKD-EPI (Panel **d**) equations with mGFR in the entire study population (*n* = 199). The dotted lines show the average of the differences between the GFR methods and the 95 % limits of agreement

Overall there was an excellent agreement between mGFR and eGFR derived from the MDRD or CKD-EPI equations as assessed by the concordance correlation coefficient as shown in Fig. 1a and b (MDRD: rho_c =0.841, 95 % CI 0.801-0.881 and CKD-EPI: rho_c =0.864, 95 % CI 0.832-0.828). There was no difference in the concordance correlation coefficient for the MDRD or CKD-EPI equations for different CKD stages. However, the difference between mGFR and eGFR derived from either equation increased proportionally to the magnitude of the mGFR, indicative of proportional bias.

The mGFR for the entire cross sectional study group was 80 ± 2.2 ml/min/1.73 m^2^. As shown in Fig. 1c and d, Bland-Altman analysis revealed a bias of -1.60 ± 16.2 ml/min/1.73 m^2^ with 95 % limits of agreement from -34.2 to 30.9 between an eGFR derived from the MDRD equation and mGFR. For an eGFR derived from the CKD-EPI equation, bias was -1.6 ± 14.6 ml/min/1.73 m^2^ with 95 % limits of agreement from -30.3 to 27.0. In the overall study population, precision (SD of the bias) was 14.6 versus 16.6 ml/min/1.73 m^2^ for the for the CKD-EPI and MDRD equations, respectively and accuracy defined by the proportion of results falling within 15 % or 30 % of mGFR values for eGFR values was P(15) 58.3 % and P(30) 90.5 % versus P(15) 51.3 % and P(30) 86.4 % for the CKD-EPI versus MDRD equations, respectively. Although precision and accuracy for eGFR values derived from the CKD-EPI compared with the MDRD equation were marginally better, these differences were not statistically significant (Table 2).

**Table 2:** Bland-Altman results together with accuracy (the proportion of results falling within 15 % or 30 % of mGFR values) and precision (the SD of the bias) for eGFR values derived from the MDRD or CKD-EPI equations with mGFR in the entire study population (*n* = 199)

	MDRD	CKD-EPI
Bias	-1.66	-1.60
SD	16.6	14.6
95 % Limits of agreement	-34.2 to 30.9	-30.3 to 27.0
P(15)	51.3 %	58.3 %
P(30)	86.4 %	90.5 %

*MDRD* modification of diet in renal disease, *CKD-EPI* Chronic Kidney Disease-Epidemiology Collaboration, *mGFR* measured glomerular filtration rate, *SD* standard deviation, *P(15)* proportion of results falling within 15 % of mGFR values, *P(30)* proportion of results falling within 30 % of mGFR values

**Fig. 2:**
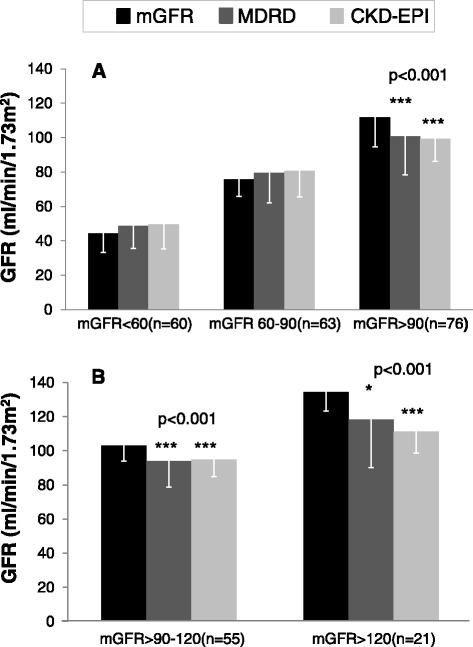
**a** eGFR values derived from the MDRD and CKD-EPI equations according to mGFR-stage 1 CKD: ≥ 90 (*n* = 60), stage 2 CKD : 60-89 (*n* = 63 ) and stage 3 CKD (*n* = 76) : < 60 ml/min/1.73 m^2^. **b** eGFR values derived from the MDRD and CKD-EPI equations according to mGFR values >90-120 (*n* = 55) and > 120 (*n* = 21) ml/min/1.73 m^2^. * *p* < 0.05, *** *p* < 0.001 vs mGFR. mGFR: Measured Glomerular Filtration Rate, MDRD: Modification of Diet in Renal Disease, CKD-EPI: Chronic Kidney Disease-Epidemiology Collaboration

As shown in Fig. 2a, for patients with mGFR >90 ml/min/1.73 m^2^ (mGFR: 112 ± 17.4), both equations underestimated mGFR to a similar extent: bias for CKD-EPI: -12.4 ± 12.2 ml/min/1.73 m^2^ (*p* < 0.001) and for MDRD: -11.0 ± 18.3 ml/min/1.73 m^2^ (*p* < 0.001). By contrast, for subjects with an mGFR < 90 ml/min/1.73 m^2^, there was a non-significant trend for both equations to overestimate mGFR. Patients with mGFR >90 ml/min/1.73 m^2^ were further stratified into those with mGFR > 90-120 (*n* = 55) or > 120 ml/min/1.73 m^2^ (*n* = 21) as shown in Fig. 2b. For patients with mGFR > 120 ml/min/1.73 m^2^ there was a very large underestimation of mGFR when eGFR values were derived from either the CKD-EPI equation: - 23.1 ± 22.8 ml/min/1.73 m^2^ (*p* < 0.001) or from the MDRD equation: -16.0 ± 28.8 ml/min/1.73 m^2^ (*p* < 0.05).

For both MDRD and CKD-EPI equations, precision tended to improve (smaller SD of the bias) as bias decreased for lower mGFR values. In contrast, accuracy as measured by both P(15) and P(30) increased for eGFR values derived from the CKD-EPI equation, but not the MDRD equation, as mGFR values increased (Table 3). Furthermore, for mGFR values > 90 ml/min/1.73 m^2^, the accuracy (P(15)), was significantly greater for eGFR values derived from the CKD-EPI compared with the MDRD equation. There was no difference the ability of the MDRD or CKD-EPI equations to correctly classify patients according to their CKD stage determined by mGFR (Table 4).

**Table 3:** Summary of bias, precision and accuracy of the MDRD and CKD-EPI equations compared to mGFR-CKD stage 1: ≥ 90, stage 2: 60-89 and < stage 3: < 60 ml/min/1.73 m^2^. Bias = eGFR-mGFR, precision = SD of the bias and accuracy = the proportion of results falling within 15 % or 30 % of mGFR values

MDRD	Bias	P(15)	P(30)
GFR >90 ml/min/1.73m^2^ (n=76)	-11.0 ± 18.3	47.4 %	89.5 %
GFR 60-90 ml/min/1.73m^2^ (n=63)	+3.8 ± 14.3	58.7 %	79.4 %
GFR <60 ml/min/1.73m^2^ (n=60)	+4.6 ± 1.0	43.3 %	83.3 %
p value	<0.001	0.06	0.57
CKD-EPI	Bias	P(15)	P(30)
GFR <90 ml/min/1.73m^2^ (n=76)	-12.4 ± 12.2	69.7 %	97.4 %
GFR <60-90 ml/min/1.73m^2^ (n=63)	+5.0 ± 11.9	57.1 %	87.3 %
GFR <60 ml/min/1.73m^2^ (n=60)	+5.3 ± 10.8	40.0 %	78.3 %
p value	<0.001	0.002	0.001

* *P* < 0.05 vs MDRD

*mGFR* measured Glomerular Filtration Rate, *CKD* chronic kidney disease, *MDRD* modification of diet in renal disease, *CKD-EPI* Chronic Kidney Disease-Epidemiology Collaboration, *P(15)* proportion of results falling within 15 % of mGFR values, *P(30)* proportion of results falling within 30 % of mGFR values

**Table 4:** Classification of the study population into CKD stages based on mGFR according to the MDRD or CKD-EPI equation

CKD stage according to mGFR	N	Classification based on the MDRD formula			Classification based on the CKD-EPI formula		
		Sensitivity	Specificity	Correctly classified	Sensitivity	Specificity	Correctly classified
≥ 90 (Stage 1)	81	64.2 %	88.98 %	78.89 %	76.5 %	84.75 %	81.41 %
60-89 (Stage 2)	58	65.5 %	70.92 %	69.35 %	58.6 %	76.6 %	71.36 %
<60 (Stage ≤ 3)	60	80 %	94.96 %	90.45 %	76.7 %	95.68 %	89.95 %

*mGFR* measured Glomerular Filtration Rate, *CKD* chronic kidney disease, *MDRD* modification of diet in renal disease, *CKD-EPI* Chronic Kidney Disease-Epidemiology Collaboration

### The influence of clinical and biochemical characteristics on the MDRD and CKD-EPI equations

**Fig. 3:**
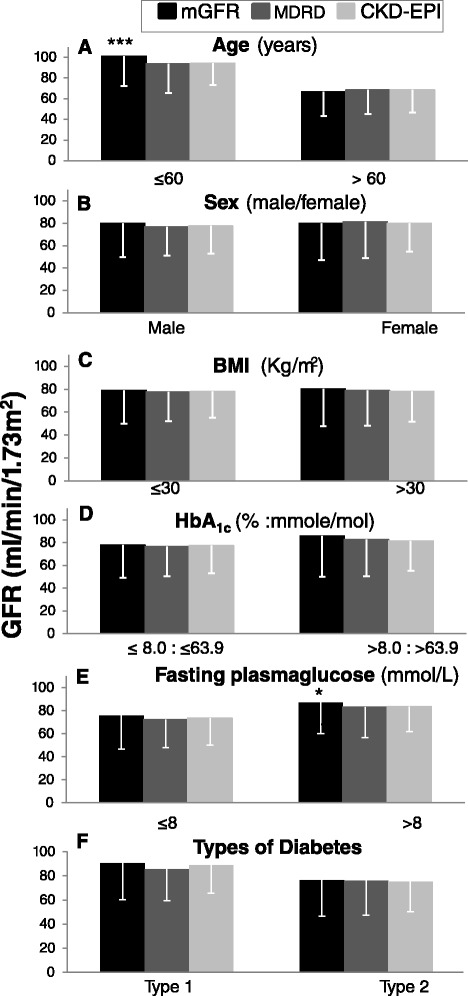
The effects of age (≤60 or > 60 years), sex (male vs female), Body Mass Index (BMI) (≤30 or > 30 kg/m^2^), glycated haemoglobin (HbA1c) (≤8 or > 8 % : ≤ 64 or > 64 mmole/mol), fasting plasma glucose (≤8 or > 8 mmol/L) or type of diabetes (type 1 or type 2) on mGFR and eGFR values derived from the MDRD and CKD-EPI equations. *** *p* < 0.001 vs mGFR, ***p < 0.001 vs mGFR for age > 60 years, * *P* < 0.05 vs mGFR for fasting plasma glucose ≤ 8 mmol/L. mGFR: measured Glomerular Filtration Rate, MDRD: Modification of Diet in Renal Disease, CKD-EPI: Chronic Kidney Disease-Epidemiology Collaboration

The effects of age (≤60 or > 60 years), sex (male vs female), BMI (≤30 or > 30 kg/m^2^), HbA1c (≤8 or > 8 % : ≤ 64 or > 64 mmole/mol), fasting plasma glucose (≤8 or > 8 mmol/L) and type of diabetes (type 1 vs 2) on mGFR and eGFR values derived from the CKD-EPI and the MDRD equations are shown in Fig. 3a-f. As expected, younger compared with older patients (Fig. 3a, *p* < 0.001) and those with higher compared with lower fasting glucose levels (Fig. 3e, *p* < 0.01) had higher mGFR values. However, age, sex, BMI, HbA1c, fasting serum glucose and type of diabetes had no influence on the difference between eGFR values derived from the MDRD or CKD-EPI equations.

**Fig. 4:**
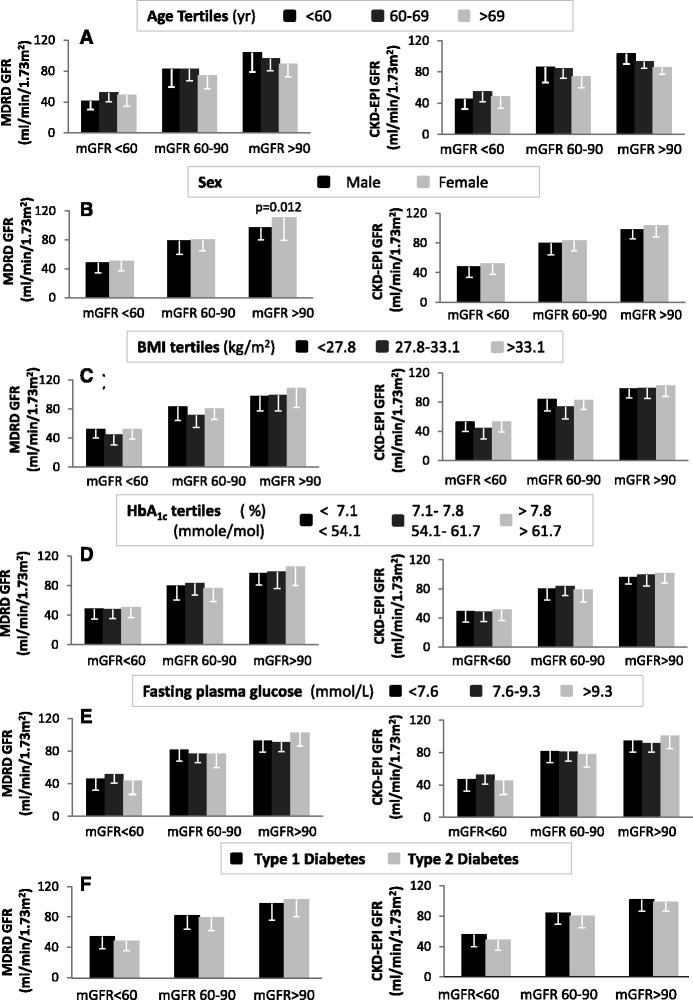
The effect of age, sex, BMI, HbA1c, fasting serum glucose levels and type of diabetes on eGFR values derived from the MDRD and CKD-EPI equations across the different strata of mGFR. *p < 0.05 vs males with mGFR > 90 ml/min/1.73 m^2^. mGFR: measured Glomerular Filtration Rate, MDRD: Modification of Diet in Renal Disease, CKD-EPI: Chronic Kidney Disease-Epidemiology Collaboration

Next we examined the effect of age, sex, BMI, HbA1c, fasting serum glucose levels and type of diabetes on eGFR values derived from the MDRD and CKD-EPI equations across the different strata of mGFR. As shown in Fig. 4b, eGFR levels estimated from the MDRD equation were significantly lower in males compared with females in the mGFR group > 90 ml/min/1.73 m^2^ but gender was not related to eGFR levels for mGFR levels < 90 ml/min/1.73 m^2^. Gender was not related to eGFR derived from the CKD-EPI equation. Age, BMI, HbA1c, fasting plasma glucose levels or type of diabetes were not related to eGFR derived from the MDRD or CKD-EPI equations across the different strata of mGFR values (Fig. 4a, c-f, respectively).

### Longitudinal analysis

**Fig. 5:**
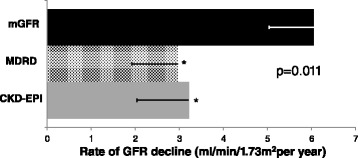
Longitudinal study of subjects with an early decline in GFR i.e., initial mGFR >60 ml/min/1.73 m^2^ and ΔmGFR ≥3.3 ml/min/1.73 m^2^ per year, ΔmGFR (based on initial and final values) was compared with ΔeGFR by the CKD-EPI and MDRD equations over a mean of 9 years (ANOVA: *p* = 0.01). *p < 0.05 vs mGFR. mGFR: measured Glomerular Filtration Rate, MDRD: Modification of Diet in Renal Disease, CKD-EPI: Chronic Kidney Disease-Epidemiology Collaboration

The clinical and biochemical characteristics of patients used for the longitudinal study are shown in Table 5. Initial and final mGFR values were 102.8 ± 6 and 54.6 ± 6.0 ml/min/1.73 m^2^, respectively over 9 years of follow up. As shown in Fig. 5, mean ΔGFR (ml/min/1.73 m^2^ per year) was 6.0 by mGFR with estimates of GFR derived by the MDRD or the CKD-EPI equations both significantly underestimating the mGFR decline (ΔGFR 3.0 and 3.2 ml/min/1.73 m^2^ per year, respectively, both *p* < 0.05 vs mGFR).

**Table 5:** Patient characteristics for the longitudinal analysis (*n* = 30). Values shown as mean ± SD or median (interquartile range)

Parameter	All (*n*=30)
Age (yr)	48.2 ± 16.0
Gender (% males)	63
Type diabetes (% T1/T2)	70/30
Duration of diabetes (yr)	17.3 ± 9.7
Height (cm)	167.7 ± 12.2
Weight (kg)	74.0 ± 16.1
BMI (kg/m^2^)	26.3 ± 4.4
mGFR (ml/min/1.73m^2^)	102.8 ± 27.9
MDRD (ml/min/1.73m^2^)	83.6 ± 27.8
CKD-EPI (ml/min/1.73m^2^)	88.3 ± 25.2
AER (μg/min)	17.8 ± (10.3, 52.5)
AER (% normo/micro/macro)	54/33/13
HbA _1c_ (%)	8.8 ± 1.3
HbA _1c_ (mmol/mol)	73
Fasting blood glucose (mmol/L)	11.4 ± 6.1
Systolic blood pressure (mmHg)	137.4 ± 17.7
Diastolic blood pressure (mmHg)	78.8 ± 9.5
Any blood pressure therapy (%)	77
ACE inhibitor therapy (%)	43
Lipid lowering therapy (%)	73
Fenofibrate	0
Retinopathy (%)	40
IHD (%)	13
CVD (%)	13
PVD (%)	10
History of macrovascular disease (%)	43

*BMI* body mass index, *mGFR* measured Glomerular Filtration Rate, *MDRD* modification of diet in renal disease, *CKD-EPI* Chronic Kidney Disease-Epidemiology Collaboration, *AER* albumin excretion rate, *Normo* normoalbuminuria, *Micro* microalbuminuria, *Macro* macroalbuminuria, *HbA1c* glycated haemoglobin, *ACE* angiotensin converting enzyme, *IHD* Ischaemic Heart Disease, *CVD* cerebrovascular disease, *PVD* peripheral vascular disease

## Discussion

We found that both the MDRD and CKD-EPI equations provided an excellent estimation of mGFR in our entire study population. However, our results confirm previous findings which have shown that both the CKD-EPI and MDRD equations significantly underestimate measured GFR to a similar extent in people with diabetes when mGFR is in the normal to high range [7, 8]. Furthermore, both equations were also found to significantly underestimate a decline in mGFR values when a group of patients were followed from normal to subnormal mGFR values over 9 years.

The only advantage that we could demonstrate for the CKD-EPI compared with the MDRD equation for estimating mGFR in the normal to high range was an improvement in accuracy. The proportion of results falling within 15 % of mGFR values > 90 ml/min/1.73 m^2^ was significantly greater for eGFR values derived from the CKD-EPI (70 %) compared with the MDRD (47 %) equation. Precision also tended be better (lower SD of the bias) for the CKD-EPI compared to the MDRD equation for mGFR values > 90 ml/min/1.73 m^2^. The clinical significance of the above finding remains to be determined. Of note, using the CD-EPI compared with the MDRD equation also did not improve the number of subjects correctly classified to CKD stage 1 or stage 2. Overall both the CKD-EPI and MDRD equations displayed poor precision. For example, the overall mGFR of the patient population was 80 ml/min/1.73 m^2^ and the SD of the bias was 16.6 ml/min/1.73 m^2^ for an eGFR derived from the MDRD equation. Hence, there would be 95 % probability of eGFR value being between 46 and 113 ml/min/1.73 m^2^. Similarly, for an eGFR derived from the CKD-EPI equation (SD of the bias 14.6 ml/min/1.73 m^2^), there would be 95 % probability of an eGFR value being between 51 and 109 ml/min/1.73 m^2^.

We attempted to address the mechanisms that explain why the CKD-EPI equation underestimates mGFR in the normal to high range to the same extent as the MDRD equation in people with diabetes. An analysis of patient characteristics such as age, sex, BMI, HbA1c levels, fasting plasma glucose values and type of diabetes, before and after stratification for mGFR values, failed to reveal any clear differences in eGFR values derived from the MDRD or CKD-EPI equations. Any relationships that we found between eGFR values derived either by the MDRD or CKD-EPI equations and the clinical characteristics of our study population are most likely explained by the expected relationship between age, glucose control and GFR. Overall, no clinical or biochemical parameter was found to account for the reasons why the CKD-EPI equation does not have a reduced bias compared with the MDRD equation in people with diabetes.

We found no relationship between BMI and estimates of GFR derived for both formulas. One study has suggested that the underestimation of mGFR by the MDRD equation is greatest in obese subjects with diabetes but this finding has been called into question as obese subjects in the above study had higher mGFR values compared to non-obese subjects [20]. Any difference in bias could therefore have been related to GFR rather than measures of obesity. In contrast, another study has found that the CKD-EPI equation did not outperform the MDRD equation in obese patients, with both equations mildly overestimating a directly measured GFR value of 56 ml/min/1.7 m^2^ [21].

High plasma glucose levels are well known to be associated with higher GFR levels [22–26]. Indeed we found that mGFR levels were high in patients with fasting BSL levels > 8 mmol/L compared to those with lower glucose levels. Recently, higher blood glucose levels have been associated with higher GFR values derived from serum cystatin C levels [27]. Whilst acute fluctuations in glucose levels may influence GFR and make the estimation of a patient’s true baseline GFR difficult to determine, we found that eGFR values derived from the CKD-EPI or the MDRD equations were similar for patients with different fasting glucose levels. Therefore, whilst plasma glucose levels can acutely influence GFR we did not find evidence to suggest that plasma glucose levels differentially affect an eGFR derived from the CKD-EPI compared with the MDRD equation.

As expected we found that that older compared with younger subjects had lower mGFR values but age had a similar effect on eGFR values from the MDRD or the CKD-EPI equations. Although we could not show that sex had a differential effect on an eGFR value from the MDRD or the CKD-EPI equations, we found that males had lower eGFR values derived from the MDRD equation than females at mGFR levels > 90 ml/min/1.73 m^2^. This may be due the MDRD equation not being able to fully account for the expected higher serum creatinine levels in males compared to females. No gender differences in eGFR values derived from the CKD-EPI formula were observed.

Although the CKD-EPI equation may perform better (less biased) than the MDRD equation in the general population, this advantage over the MDRD equation for estimating GFR is not as apparent in people with diabetes. In a secondary analysis of the population originally used to validate the CKD-EPI equation, the performance of the CKD-EPI equation was examined according to level of GFR and clinical characteristics (28 % with diabetes). The overall conclusion of the study was that the CKD-EPI compared with the MDRD equation substantially improves bias in people with and without diabetes [5].

However, in the above study, eGFR values calculated from the CKD-EPI equation underestimated measured GFR values by 4.6 compared to only 1.3 ml/min/1.73 m^2^, in people with and without diabetes, respectively. This greater bias in people with diabetes was also more pronounced when GFR was > 90 ml/min/1.73 m^2^. In particular, for participants with diabetes and GFR >90 ml/min/1/73 m^2^, eGFR values derived from the CKD-EPI and MDRD equations underestimated measured GFR by 12.3 and 19.1.ml/min/1.73 m^2^, respectively [5]. Reports of a lack of improvement in the underestimation of GFR with the CKD-EPI equation compared with the MDRD equation in people with diabetes and normal to high GFR levels may therefore not be an unexpected finding [6–10].

Of relevance to our findings, a decrease in the performance of the CKD-EPI equation is being increasingly recognised in people with diabetes compared to those without diabetes. Camargo et al found that CKD-EPI equation was less accurate in people with type 2 diabetes when compared to healthy individuals [10]. One of the limitations of that study was that serum creatinine levels measured by the Jaffe reaction are possibly subject to interference from plasma glucose levels and other chromogens [28].

We have also recently demonstrated that when serum creatinine is assayed by the gold standard enzymatic method, the CKD-EPI equation also has greater bias in Indigenous Australians with diabetes compared with those without diabetes, especially in those with normal to high renal function. In the above study, when mGFR was > 90 ml/min/1.73 m^2^, an eGFR derived from the CKD-EPI or MDRD equations underestimated measured GFR by a similar extent, 7.4 and 7.2 ml/min/1.73 m^2^, respectively [9]. One of the limitations of the above studies was that they were cross sectional in design and did not specifically examine the performance of the CKD-EPI equation according to the clinical characteristics of participants that had a direct measurement of GFR.

Our findings have significant implication for estimating renal function trajectories in people with diabetes. Use to the CKD-EPI equation did not improve the underestimation of the rate of decline in GFR from normo-filtration to hypo-filtration that was observed when eGFR was derived from the MDRD equation. This significant underestimation of measured GFR trajectories by the MDRD equation in subjects with type 1 diabetes has been previously reported by our group [15]. Currently, two large longitudinal studies have compared rates of estimated with measured GFR decline over a relatively short follow up interval of 3 to 4 years [8, 29].

One study has assessed the agreement of 15 creatinine based formulas for estimating GFR with measured GFR values (iohexol plasma clearance) in 600 patients with type 2 diabetes with a baseline mGFR of 101 ml/min/1.73 m^2^ in a cross-sectional analysis and in a longitudinal analysis for 449 patients that had serial mGFR measurements over 4 years (rate of GFR decline -3.37 ml/min/1.73 m^2^). The overall agreement between measured and all estimates of GFR was poor in both the cross-sectional and the longitudinal analysis. In the entire study population all 15 formulas underestimated the rate of mGFR decline [8].

In a study of 997 patients with type 1 diabetes involved in the Diabetes Control and Complications Trial/Epidemiology of Diabetes Interventions and Complications (DCCT/EDIC) study who had direct measurements of GFR (^125^Iothalamate urinary clearance) over a mean interval duration of 3.1 years, changes in eGFR derived from the CKD-EPI equation did not compare favorably with changes in mGFR. Although bias was small, the correlation and precision of eGFR values derived from the CKD-EPI formula were poor when compared to mGFR values [29].

Cystatin C has been proposed as an alternative endogenous marker of GFR to creatinine. The improvement in accuracy for estimates of GFR based on cystatin C compared with creatinine remains to be fully established. Early studies based on estimating GFR from the reciprocal value of serum cystatin C suggested that cystatin C was less biased and provided a more accurate method for estimating GFR values than creatinine based methods when directly measured GFR is in the normal to hyperfiltering range [12, 30–32]. The very large under estimation of mGFR values that we observed with an eGFR derived from the CKD-EPI equation emphasizes that there is a need to improve estimates of GFR when true GFR is in the hyperfiltering range.

However, more recent studies using cystatin C values harmonized against an international reference standard and the CKD-EPI 2102 cystatin equation have failed to show that that estimating GFR based on cystatin C compared with creatinine provides a substantial improvement in accuracy [33]. The use of GFR estimating equations based on a combination of serum cystatin C and creatinine values may provide a more accurate way of estimating GFR in routine clinical practice [33–35]. Unfortunately, we did not have cystatin C values measured using a harmonized assay to derive cystatin-eGFR or cystatin-creatinine-eGFR values to allow a comparison of bias, precision and accuracy with creatinine-eGFR or values in the current study.

The limitations of this study, including the relatively small number of participants, especially in the longitudinal study and that the longitudinal study included only two estimates of GFR for each participant are acknowledged. Furthermore, in our longitudinal study, stored serum samples for re-assay were not available and estimates of eGFR were based on creatinine levels that were stored on our data base, and that were originally measured by the Jaffe reaction. An attempt to harmonise theses historical results with an enzymatic method was attempted but it is appreciated that this approach has its limitations. Possibly, longer studies that examine the relationship between eGFR, measured GFR and multiple clinical and biochemical variables at multiple time points in a prolonged longitudinal study of people with and without diabetes will help to delineate the reasons why the CKD-EPI equation preferentially underestimated GFR in people with diabetes .

## Conclusions

In conclusion, in this study, both the CKD-EPI and MDRD equations underestimate mGFR levels > 90 ml/min/1.73 m^2^ by approximately 11 ml/min/1.73 m^2^. For mGFR values > 120 ml/min/1.73 m^2^, the degree of underestimation of GFR derived by the CKD-EPI equation was even more pronounced (23 ml/min/1.73 m^2^) with no improvment in underestimation compared with the MDRD equation. The mechanisms responsible for the failure of the CKD-EPI equation to negate underestimation of true GFR values in people with diabetes remains unknown and does not appear to be explained by patient characteristics such as age, sex, BMI, HbA1c or fasting plasma glucose levels. We also found that using the CKD-EPI compared with the MDRD equation does increase the number of subjects with diabetes that are correctly classified with having CKD stage 1. The underestimation of mGFR by the CKD-EPI equation has significant implications for correctly identifying patients with diabetes and GFR values in the normal and hyperfiltering range and accurately following a possible subsequent decline in GFR. There is clearly a need to improve estimates of GFR in people with diabetes who have GFR values > 90 ml/min/1.73 m^2^.

## Abbreviations

BMI: body mass index
CKD: Chronic Kidney Disease
CKD-EPI: Chronic Kidney Disease-Epidemiology Collaboration
Concordance Correlation Coefficient rho_c: the proportion of results falling within 15 % or 30 % of measured GFR values, (P15) and (P30), respectively
CVD: cerebrovascular disease
DTPA: ^99m^Tc-diethylene-triamine-pentaacidic acid
eGFR: estimated gomerular filtration rate
HbA1c: Glycated haemoglobin
IDMS: Isotope Dilution Mass Spectrometry
IHN: Angiotensin Converting Enzyme, Ischaemic Heart Disease
MDRD: modification of diet in renal disease
mGFR: measured glomerular filtration rate
PVD: Peripheral Vascular Disease
SD: standard deviation
